# Defining the Therapeutic Range for Adalimumab and Predicting Response in Psoriasis: A Multicenter Prospective Observational Cohort Study

**DOI:** 10.1016/j.jid.2018.07.028

**Published:** 2019-01

**Authors:** Nina Wilkinson, Teresa Tsakok, Nick Dand, Karien Bloem, Michael Duckworth, David Baudry, Angela Pushpa-Rajah, Christopher E.M. Griffiths, Nick J. Reynolds, Jonathan Barker, Richard B. Warren, A. David Burden, Theo Rispens, Deborah Stocken, Catherine Smith, BSTOP study group, PSORT consortium

**Affiliations:** 1 Institute of Health and Society, Faculty of Medical Sciences, Newcastle University, Newcastle-upon-Tyne, UK; 2 St John’s Institute of Dermatology, School of Basic and Medical Biosciences, Faculty of Life Sciences and Medicine, King’s College London, London, UK; 3 St. John’s Institute of Dermatology, Guy’s and St Thomas’ NHS Foundation Trust, London, UK; 4 Department of Immunopathology, Sanquin Research and Landsteiner Laboratory, Amsterdam, The Netherlands; 5 Dermatology Centre, Salford Royal NHS Foundation Trust, The University of Manchester, Manchester Academic Health Science Centre, NIHR Manchester Biomedical Research Centre, Manchester, UK; 6 Dermatological Sciences, Institute of Cellular Medicine, Medical School, Newcastle University, and Department of Dermatology, Royal Victoria Infirmary, Newcastle Hospitals NHS Foundation Trust, Newcastle upon Tyne, UK; 7 Institute of Infection, Immunity and Inflammation, University of Glasgow, UK; 8 Leeds Institute of Clinical Trials Research, University of Leeds, Leeds, UK

**Keywords:** ADA, anti-drug antibody, BADBIR, British Association of Dermatologists Biologic Interventions Registry, BSTOP, Biomarkers of Systemic Treatment Outcomes in Psoriasis, CI, confidence interval, IBD, inflammatory bowel disease, IMID, immune-mediated inflammatory disease, PASI, Psoriasis Area and Severity Index, PASI75, 75% improvement in baseline Psoriasis Area and Severity Index, PASI90, 90% improvement in baseline Psoriasis Area and Severity Index, RA, rheumatoid arthritis

## Abstract

Biologics have transformed management of inflammatory diseases. To optimize outcomes and reduce costs, dose adjustment informed by circulating drug levels has been proposed. We aimed to determine the real-world clinical utility of therapeutic drug monitoring in psoriasis. Within a multicenter (n = 60) prospective observational cohort, 544 psoriasis patients were included who were receiving adalimumab monotherapy and had at least one serum sample and Psoriasis Area and Severity Index (PASI) score available within the first year. We present models giving individualized probabilities of response for any given drug level: a minimally effective drug level of 3.2 μg/ml discriminates responders (PASI75 indicates 75% improvement in baseline PASI) from nonresponders, and gives an estimated PASI75 probability of 65% (95% confidence interval = 60–71). At 7 μg/ml, PASI75 probability is 81% (95% CI = 76–86); beyond 7 μg/ml, the drug level/response curve plateaus. Crucially, drug levels are predictive of response 6 months later, whether sampled early or at steady state. We confirm serum drug level to be the most important factor determining treatment response, highlighting the need to take drug levels into account when searching for biomarkers of response. This real-world study with pragmatic drug level sampling provides evidence to support the proactive measurement of adalimumab levels in psoriasis to direct treatment strategy, and is relevant to other inflammatory diseases.

## Introduction

Biologic therapies have transformed the treatment paradigm in immune-mediated inflammatory diseases (IMIDs). Complete disease remission is now achievable in people with psoriasis, rheumatoid arthritis (RA), and inflammatory bowel disease (IBD), where inhibition of the inflammatory cytokine tumor necrosis factor-α (TNF- α) remains the first-line biologic strategy. However, there are wide variations in response, with a significant number of patients not responding (primary treatment failure) or losing response over time (secondary treatment failure) (Garces et al., 2013, Yanai and Hanauer, 2011). Some of this heterogeneity may be explained by differences in the amount of drug available at the target tissue, which in turn is influenced by adherence and pharmacokinetic covariates such as weight and drug immunogenicity (formation of antidrug antibodies [ADAs]). Therapeutic drug monitoring using measurement of serum drug levels (a proxy for tissue levels) and/or ADAs thus holds potential to optimize management, and a strong correlation between TNF inhibitor serum trough levels, ADAs, and treatment response has been described in IBD, RA, and psoriasis (Baert et al., 2003, Chen et al., 2015, Lecluse et al., 2010). Indeed, a recent study using adalimumab clinical trial data in 1,212 psoriasis patients reported that responders at 16 weeks had higher adalimumab concentrations than nonresponders (6.3 vs. 2.2 μg/ml). Bodyweight was a significant covariate in the pharmacokinetic model, and the presence of ADAs resulted in lower adalimumab exposure and efficacy (Mostafa et al., 2017).

Effective therapeutic drug monitoring requires the definition of a therapeutic range, and although parameters for serum adalimumab levels have been proposed in the context of several IMIDs (Menting et al., 2015, Pouw et al., 2015, Roblin et al., 2014, Yarur et al., 2016), these have not yet been validated in psoriasis patients. Furthermore, the utility of drug level as a predictor of subsequent response has not been investigated in psoriasis other than in a previous preliminary study by our group (Mahil et al., 2013). Defining clinical outcomes in IBD and RA is inherently challenging—often relying on composite indices comprising patient-reported criteria and nonspecific biochemical markers. Psoriasis provides a disease model less encumbered by such issues, because treatment response can be visually observed and easily quantified. Furthermore, biologics are generally used as monotherapy (whereas patients with IBD and RA are often co-prescribed immunosuppressants such as methotrexate, known to reduce the formation of ADAs). Here, we capitalize on a real-world bioresource from a large multicenter cohort study, Biomarkers of Systemic Treatment Outcomes in Psoriasis (BSTOP), within the UK pharmacovigilance registry British Association of Dermatologists Biologic Interventions Registry (BADBIR), to investigate the clinical utility of therapeutic drug monitoring as applied to the exemplar TNF inhibitor adalimumab. This work is particularly timely with the imminent release of adalimumab biosimilar products to market, because optimizing outcomes may deliver comparable efficacy to newer biologics, but at significantly lower cost. We explore the relationship between drug levels and treatment response, accounting for individual patient characteristics to determine (i) the adalimumab therapeutic range (i.e., both the minimal effective drug level and the drug level beyond which response plateaus) and (ii) whether drug level predicts longer-term response. Given the nature of this real-world dataset, findings are generalizable to clinical practice.

## Results

### Description of the cohort and patient characteristics

**Figure 1:**
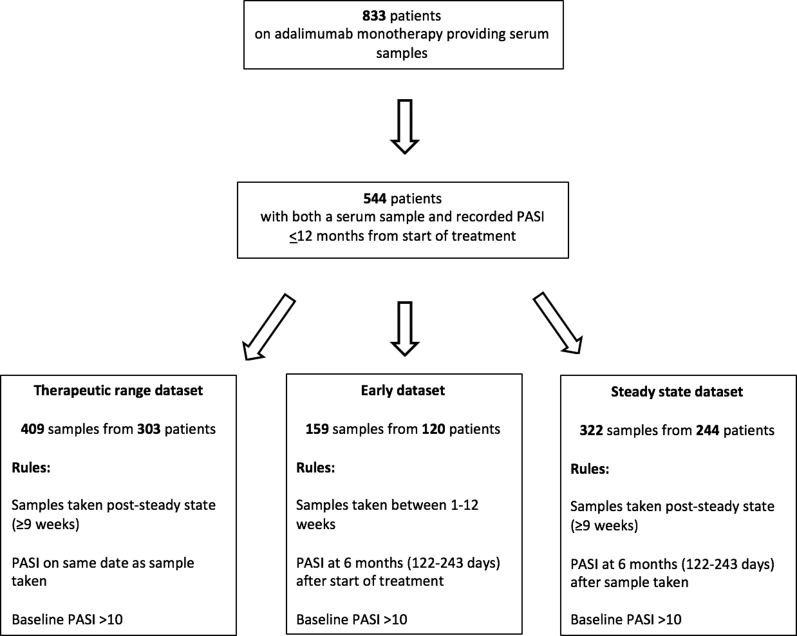
**Flow diagram of patients and samples.** Flow diagram showing the rules applied to derive the three datasets. PASI, Psoriasis Area and Severity Index.

At the time of the data cut in April 2017, 2028 patients were currently or previously receiving adalimumab monotherapy within the BSTOP cohort. Of these, 1,242 consented to give longitudinal serum samples; within this, serum samples were actually collected from 833 patients. Baseline characteristics were similar between those providing and not providing samples (Table 1, and see Supplementary Table S1 online). Of the 833 patients providing serum samples, 544 patients also had Psoriasis Area and Severity Index (PASI) data within 12 months of starting adalimumab (Figure 1). These 544 patients were included in the analysis (Table 1), and of these, 375 (69%) were biologic naïve.

**Table 1:** Summary statistics for the full cohort, therapeutic range dataset, early dataset, and steady state dataset^1^

Covariate	Full Cohort (n = 544 patients with 961 samples)		Therapeutic Range Dataset (n = 303 patients with 409 samples)		Early Dataset (n = 120 patients with 159 samples)		Steady State Dataset (n = 244 patients with 322 samples)	
	Mean (SD)	Complete Data, n (%)	Mean (SD)	Complete Data, n (%)	Mean (SD)	Complete Data, n (%)	Mean (SD)	Complete Data, n (%)
Baseline PASI	13.5 (6.7)	495 (91.0)	15.9 (5.6)	303 (100.0)	16.2 (6.4)	120 (100.0)	15.9 (5.6)	244 (100.0)
Height (cm)	172.3 (10.3)	520 (95.6)	172.0 (10.1)	295 (97.4)	172.4 (9.3)	114 (95.0)	172.3 (10.3)	239 (98.0)
Weight (kg)	90.9 (20.4)	471 (86.6)	92.3 (20.7)	277 (91.4)	92.3 (22.2)	106 (88.3)	92.9 (21.1)	223 (91.4)
Waist (cm)	102.1 (15.6)	443 (81.4)	103.0 (16.0)	266 (87.8)	103.2 (16.9)	103 (85.8)	103.8 (15.7)	214 (87.7)
BMI (kg/m^2^)	30.8 (6.7)	465 (85.5)	31.3 (7.2)	274 (90.4)	31.2 (7.3)	106 (88.3)	31.3 (7.0)	221 (90.6)
Age (years)	44.3 (12.2)	544 (100.0)	44.0 (12.3)	303 (100.0)	43.8 (12.4)	120 (100.0)	44.1 (12.2)	244 (100.0)
Disease duration (years)	22.0 (12.0)	498 (91.5)	21.5 (12.4)	282 (93.1)	20.8 (11.5)	104 (86.7)	21.1 (11.8)	233 (95.5)
	**n (%)**		**n (%)**		**n (%)**		**n (%)**	
Ethnicity, white	484 (89.0)	544 (100.0)	272 (89.8)	303 (100.0)	103 (85.8)	120 (100.0)	216 (88.5)	244 (100.0)
Sex, male	338 (62.1)	544 (100.0)	191 (63.0)	303 (100.0)	80 (66.7)	120 (100.0)	161 (66.0)	244 (100.0)
Inflammatory arthritis	109 (23.5)	464 (85.3)	62 (22.6)	274 (90.4)	27 (26.2)	103 (85.8)	54 (24.1)	224 (91.8)
Ever smoked	298 (56.7)	526 (96.7)	172 (57.9)	297 (98.0)	66 (57.9)	114 (95.0)	141 (58.5)	241 (98.8)
Palm psoriasis	87 (16.9)	515 (94.7)	46 (16.0)	288 (95.0)	21 (19.4)	108 (90.0)	38 (16.4)	232 (95.1)
Biologic naive	375 (68.9)	544 (100.0)	237 (78.2)	303 (100.0)	97 (80.8)	120 (100.0)	189 (77.5)	244 (100.0)

Abbreviations: BMI, body mass index; PASI, Psoriasis Area and Severity Index; SD, Standard Deviation.

1 Summaries for the therapeutic range, early, and steady state datasets are restricted to patients with baseline PASI > 10. Height, waist, and body mass index measurements provided for information only; weight used in modeling.

The demographics and clinical characteristics of the cohort were consistent with severe disease (predominantly male, with an elevated body mass index and mean baseline PASI of 13.5 (standard deviation = 6.7) (Table 1)). Drug levels were sampled according to standard clinical care (median time from last dose = 7 days, interquartile range = 6–10 days, range = 0–14 days, data available on n = 349 samples), giving a mean drug level of 5.83 μg/ml (standard deviation = 3.86, range = 0.01–22 μg/ml).

**Figure 2:**
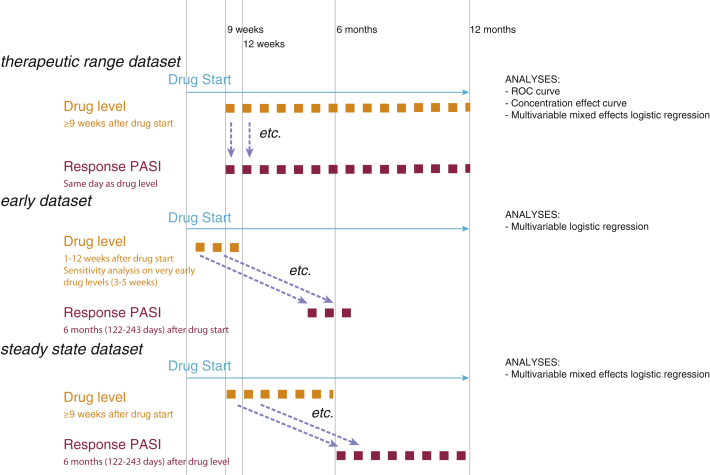
**Timeline of drug levels and response in each dataset.** Timeline showing when drug level and response were measured in each of the three datasets. In the therapeutic range dataset, response was measured on the same day as drug level. The other two datasets were derived to investigate use of drug levels to predict response 6 months later: in the early dataset, response was measured at 6 months after start of treatment; in the steady state dataset, response was measured 6 months after drug level. Statistical analyses conducted using each dataset are also shown. ROC, receiver operating characteristic.

As Figure 1, Figure 2 show, three datasets were derived: a therapeutic range dataset to investigate the relationship between drug levels and same-day response, an early dataset to investigate the relationship between drug levels taken before 12 weeks and the 6-month response, and a steady state dataset to investigate the relationship between drug levels taken any time after 9 weeks and response 6 months later.

### Defining the therapeutic range

#### Drug level discriminates responders from nonresponders

**Figure 3:**
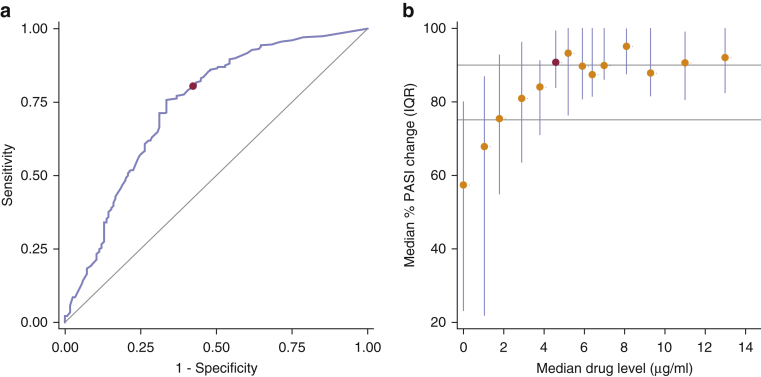
**(a) Empirical ROC curve. (b) Concentration effect curve.** (**a**) Empirical ROC curve for PASI75 response. Cutpoint (red dot) chosen to provide a minimum sensitivity of 80%. (**b**) Concentration effect curve of median percentage change in PASI against median drug level. These summaries are calculated for approximately equally sized groups of observations (between 23 and 52) having similar drug levels. Vertical bars: interquartile range (IQR); grey horizontal lines: indicators of PASI75 and PASI90 response; red dot: drug level beyond which clinical response plateaus. IQR, interquartile range; PASI, Psoriasis Area and Severity Index; PASI75, 75% improvement in baseline PASI; ROC, receiver operating characteristic.

Using the therapeutic range dataset (drug levels with PASI recorded on the same day), empirical receiver operating characteristic curves (Menting et al., 2015) were generated for all three outcomes. For a 75% improvement in baseline PASI score (PASI75), drug level discriminated responders from nonresponders with an area under the curve of 0.74 (95% confidence interval [CI] = 0.68–0.79) (Figure 3a, Table 2). A lower limit of 3.2 μg/ml identified patients achieving PASI75 with our preset minimum sensitivity of 80% (red dot on Figure 3a; specificity = 58%, overall classification accuracy = 73%). This drug level showed comparable sensitivity for the secondary outcomes of 90% improvement in baseline PASI score (PASI90) (82%) and absolute PASI of 1.5 or less (PASI≤1.5) (85%), but specificity and overall classification accuracy were lower (see Supplementary Table S2 online).

**Table 2:** Diagnostic accuracy of the therapeutic range for PASI75 response

	Drug Levels and Response (Same Day)		Drug Levels as a Predictor of Subsequent Response (6 Months)			
			Early		Steady State	
Cutpoint^1^ (μg/ml)	3.2	7	3.2	7	3.2	7
Sensitivity	80.28	38.38	86.61	40.18	77.46	39.44
Specificity	57.60	84.80	44.68	74.47	55.96	84.40
Overall classification accuracy	73.35	52.57	74.21	50.31	70.19	54.66
Positive predictive value	81.14	85.16	78.86	78.95	77.46	83.17
Negative predictive value	56.25	37.72	58.33	34.31	55.96	41.63
AUC (95% CI)	0.74 (0.68–0.79)		0.70 (0.59–0.80)		0.72 (0.66–0.78)	
Response rate: all samples	69.44		70.44		66.15	
Response rate: samples with drug level < cutpoint^2^	43.75	62.28	41.67	65.69	44.04	58.37
Response rate: samples with drug level ≥ cutpoint^2^	81.14	85.16	78.86	78.95	77.46	83.17
Probability of response^3^ (95% CI)	65 (60–71)	81 (76–86)	61 (51–70)	78 (71–85)	77 (71–83)	64 (58–70)

Analyses are based on 409 samples from 303 patients for the therapeutic range, on 159 samples from 120 patients for the early samples, and on 322 samples from 244 patients for the steady state dataset.

Abbreviations: AUC, area under the curve; CI, confidence interval.

1 A cutpoint of 3.2 indicates that samples with a drug level of 3.2 μg/ml or greater are predicted to correspond with response.

2 Response rates for samples above and below cutpoints are equivalent to positive predictive value and to 1 – negative predictive value, respectively.

3 Expressed as percentage. Derived from the final multivariable models given in Table 3.

#### Likelihood of response increases with increasing drug level and then plateaus

Using the therapeutic range dataset (drug levels with PASI recorded on the same day), a descriptive concentration effect curve (Menting et al., 2015) was next generated to confirm that clinical response increases with increasing drug level, then plateaus for groups with median drug level of 4.6 μg/ml or greater (red dot on Figure 3b), corresponding to a percent PASI change of 90.7% (IQR = 83.7–99.4, range = 16.2–100). However, the interquartile ranges on this curve show variability in response, likely caused by other clinical and confounding factors including ADAs, time from last dose, sex, age, and disease duration.

#### Selecting an upper limit of the therapeutic range taking other covariates into account

**Figure 4:**
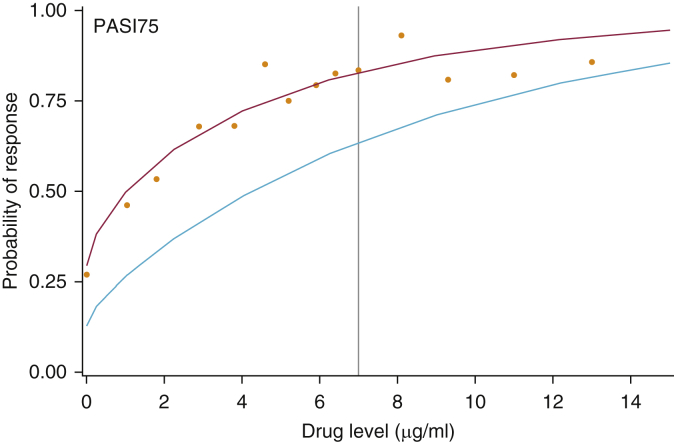
**Probability of PASI75 based on same-day drug level (therapeutic range dataset).** Probability of response is split by ethnicity (red = white ethnicity, teal = all other ethnicities). The grey vertical line is at a drug level of 7 μg/ml, where there is at least 80% probability of response on average for all patients. This line crosses the red curve for patients of white ethnicity at a probability of response greater than 80%, but the probability is lower for the non-white group (teal line).The orange dots indicate the proportion of patients per group achieving PASI75. The groups are calculated in the same way as for the concentration effect curve in Figure 2b, and they are not split by other covariates. The probabilities are marginal predicted means because of the inclusion of a random effect in the model. Similar curves are seen for probability of PASI75 in the other datasets (early and steady state). PASI75, 75% improvement in baseline PASI.

To take clinical and confounding covariates into account, multivariable mixed effects logistic regression modelling was carried out using the therapeutic range dataset. The results were consistent with the empirical analysis: for the primary outcome of PASI75, the best-fitting model included (transformed) drug level and ethnicity as covariates (Table 3, and see Supplementary Table S3 online), and the probability of PASI75 response plateaued with increasing drug level, supporting the concept of an upper-bounded therapeutic range (Figure 4). We selected 7 μg/ml as the upper limit of the therapeutic range because this achieves our minimum stipulated 80% probability of response (81%, 95% CI = 76–86) (Figure 4), whereas the drug level at which the concentration effect curve appeared to plateau (4.6 μg/ml) has a lower probability of response (73%, 95% CI = 68–77).

**Table 3:** Final multivariable models for PASI75 response based on drug level and additional covariates (same-day response – therapeutic range dataset; response 6 months later – early dataset and steady state dataset)

Therapeutic Range Dataset (Mixed Effects Logistic Regression Model)								
	Covariate	Coefficient (SE)	95% CI	OR (95% CI)	*P*-Value	Marginal/Conditional Pseudo *R*^2^	Number of Samples	Number of Responders (% of Samples)
PASI75	Sqrt (drug level)	1.10 (0.20)	0.69–1.50	2.99 (2.00–4.46)	<0.001	0.25/0.38	409 samples from 303 patients	284 (69.44)
	Ethnicity, white	1.15 (0.46)	0.24–2.06	3.17 (1.28–7.85)	0.013			
**Early Dataset (Logistic Regression Model)**								

	**Covariate**	**Coefficient (SE)**	**95% CI**	**OR (95% CI)**	***P*-Value**	**Pseudo *R***^**2**^	**Number of Samples**	**Number of Responders (% of Samples)**

PASI75	Sqrt (drug level)	1.00 (0.26)	(0.49–1.52)	2.73 (1.63–4.57)	<.001	0.10	159 samples on 120 patients	112 (70.44)
	Ethnicity, white	1.05 (0.51)	(0.06–2.04)	2.86 (1.06–7.72)	.039			
**Steady State Dataset (Mixed Effects Logistic Regression Model)**								

	**Covariate**	**Coefficient (SE)**	**95% CI**	**OR (95% CI)**	***P*-Value**	**Marginal/Conditional Pseudo *R***^**2**^	**Number of Samples**	**Number of Responders (% of Samples)**

PASI75	Sqrt (drug level)	1.02 (0.21)	0.60–1.44	2.78 (1.83–4.24)	<.001	0.16/0.50	322 samples on 244 patients	213 (66.15)

Abbreviations: CI, confidence interval; OR, odds ratio; PASI75, 75% improvement in baseline PASI; SE, standard error; Sqrt, square root.

For the PASI90 and PASI≤1.5 outcomes, drug level remained the most important determinant of response. Additional covariates appear significant for PASI90 (baseline PASI, treatment duration, and biologic-naïve status) and PASI≤1.5 (sex and biologic-naïve status). However, this should be treated with caution given the small number of patients achieving PASI90 and PASI≤1.5 (see Supplementary Tables S3 and S4 and Supplementary Figure S1 online).

### Using drug level to predict subsequent response

#### Early drug levels predict response at 6 months

To determine whether drug levels indicate response status at later time points, multivariable logistic regression modeling was carried out using the early dataset (drug levels taken between 1 and 12 weeks, with PASI recorded at 6 months). For the primary outcome of PASI75, independent predictors were (transformed) drug level and ethnicity (Table 3, and see Supplementary Table S5 online). These same covariates were included in the final PASI90 model, and drug level and baseline PASI were included in the PASI≤1.5 model (see Supplementary Table S4 online). Similar to the analysis using the therapeutic range dataset (exploring the relationship between drug levels and response on the same day), the probability of response at 6 months increases with increasing early drug level (PASI75: Table 3, and see Supplementary Figure S2a; PASI90 and PASI≤1.5: see Supplementary Table S4 and Supplementary Figures S2b and c). The upper limit of 7 μg/ml (defined in the therapeutic range dataset) (Figure 4) corresponds to a 78% (95% CI = 71–85) probability of 6-month response using early drug levels (Table 2, and see Supplementary Figure S2a). We also performed a sensitivity analysis by fitting the model to very early samples (4 weeks ± 1 week after treatment initiation), given our pilot data showing that higher adalimumab levels in responders versus nonresponders were detectable at 4 weeks (Mahil et al., 2013), and acknowledging the overlap between our early dataset and steady state dataset. We found a similar relationship between drug levels and PASI75 response (see Supplementary Table S6 online).

#### Steady state drug levels predict response 6 months later

Finally, we explored whether steady state drug levels in patients established on therapy could predict treatment outcomes 6 months later. Multivariable mixed effects logistic regression modeling was carried out using the steady state dataset (drug levels taken at least 9 weeks after treatment start, with PASI recorded 6 months later) (Table 3, and see Supplementary Table S7 online). Again, for the primary outcome of PASI75, (transformed) drug level was the single most important predictor of response (odds ratio [square root of drug level] = 2.78, 95% CI = 1.83–4.24, *P* < 0.001) (Table 3), with increasing probability of response with increasing drug level (see Supplementary Figure S3a). This relationship between drug level and response was also seen using the PASI90 and PASI≤1.5 outcomes (see Supplementary Table S4 and Supplementary Figure S3b and c); the covariates palm psoriasis and biologic-naïve status were also significant for these outcomes, respectively.

### Clinical utility of the therapeutic range

Table 2 gives the standard estimates of clinical utility for our therapeutic range for PASI75 (3.2–7.0 μg/ml). This has comparable diagnostic accuracy whether used to determine response on the same day as the drug level, or response 6 months later. Bearing our therapeutic range in mind, 72 of 125 (57.60%) samples with a corresponding response less than PASI75 had a drug level below 3.2 μg/ml, and 69 of 171 (40.35%) samples with a corresponding PASI90 response had a drug level greater than or equal to 7 μg/ml. This suggests that a significant proportion of the cohort would benefit from treatment modification.

## Discussion

### Key results

In the largest real-world, multicenter cohort across any IMID to date, we determined the therapeutic range for adalimumab in moderate to severe psoriasis and calculated probabilities of response for any given drug level for multiple outcomes (PASI75, PASI90, and absolute PASI≤1.5). We also show that drug levels can be used to predict response at later time points, whether taken early in the treatment course or at steady state. A minimal effective circulating drug level of 3.2 μg/ml distinguishes PASI75 responders from nonresponders, and a target drug level of 7 μg/ml provides an 80% probability of achieving a PASI75 response. As expected, there is a lower probability (51%) of achieving higher disease clearance (PASI90) at the same target drug level. Measurement of ADA provides no additional clinical utility, presumably because of the correlation between drug levels and ADAs. These key findings support the practical utility of measuring drug levels at routine clinical visits (irrespective of timing in relation to drug administration, and despite not being at trough levels) and provide drug level thresholds at which to consider changes in adalimumab treatment.

### Context and clinical implications

Our findings are consistent with the only other study conducted in psoriasis, which was dual-center and reported a therapeutic range of 3.51 to 7.00 μg/ml (Menting et al., 2015) but did not take into account clinical or other covariates such as ADAs, nor comment on clinical utility. This work builds on our previous pilot data showing that adalimumab levels at 4 weeks were significantly higher in responders than nonresponders (Mahil et al., 2013). Very few studies across any IMID have paid attention to early drug levels, and to our knowledge, only one small study in IBD (Baert et al., 2014) has looked at early adalimumab levels as a predictor of subsequent treatment response. This may open up a powerful clinical opportunity to optimize therapy before drug levels have reached steady state, well ahead of clinical relapse. Furthermore, it is significant that drug level at steady state (≥9 weeks) is also associated with response 6 months later, suggesting that there is potential to optimize therapy even once patients are established on treatment. Our results may be generalizable across other IMIDs, given comparable therapeutic ranges for adalimumab reported in RA (5–8 μg/ml) (Pouw et al., 2015) and IBD (5.0–5.9 μg/ml or 4.9–7.5 μg/ml [Roblin et al., 2014, Yarur et al., 2016]) and comparable mean levels in ankylosing spondylitis (Kobayashi et al., 2012).

A minimal effective drug level indicates the threshold below which treatment should be modified (dose escalation or treatment switch). Such an approach has been tested in IBD, with the Trough Level Adapted Infliximab Treatment (TAXIT) trial showing similar remission rates in IBD patients on adjusted infliximab dosing based on drug level, but with fewer flares than the conventional approach (Vande Casteele et al., 2015). An upper limit of the therapeutic range identifies a patient population that might benefit from dose minimization. This has been simulated in an RA cohort and although cost effective, it led to a reduction in quality-adjusted life years for at least a quarter of patients (Krieckaert et al., 2015). By comparison, our modeling approach may have an advantage in allowing for individualized prediction of response. Indeed, our data indicate that both the stringency of the outcome (for example PASI75 or PASI90) and the threshold set for the probability of response, need to be considered when defining the upper limit of the therapeutic range (and therefore the drug level chosen to implement dose minimization). This will help optimize cost effectiveness and minimize the proportion of patients subjected to inappropriate dose reduction.

From a biological perspective, the finding that the clinical response rate plateaus beyond a certain drug level likely reflects the point at which most of the TNF in psoriatic skin is neutralized by adalimumab. In turn, this may indicate that in patients for whom clinical response plateaus at a lower drug level, alternative non-TNF pathways possibly play a greater role in driving their psoriasis.

The finding that biologic-naïve status and ethnicity may predict longer-term response requires further validation, because these covariates were not consistent across outcomes. Our data suggest that although biologic-naïve status does not appear to be important for achieving PASI75, it does appear to be influential for achieving clearance (PASI90 or PASI≤1.5). This is consistent with existing evidence that biologic-naïve status may influence outcome (National Institute for Health and Care Excellence, 2018).

Our findings related to ethnicity should be treated with particular caution, because very few patients within the study cohort were of non-white ethnicity. Across the BADBIR cohort as a whole, we found non-white ethnicity to be associated with a reduced likelihood of response to biologics up to 1 year (Warren et al., 2018).

Finally, we have confirmed serum drug level to be the single most important factor determining treatment response, whether sampled a few weeks after treatment initiation or at steady state. This underpins the importance of taking drug levels into account when searching for biomarkers and mechanisms of treatment response, such as genetic factors. Indeed, the need to incorporate a richer set of clinical information, such as seropositivity and disease duration, was recently highlighted in an innovative crowdsourced assessment of the common genetic contribution to predicting TNF antagonist treatment response in RA (Sieberts et al., 2016).

### Strengths and limitations

A key strength of this study is high external validity, because more than 50% of all UK psoriasis patients taking biologics are registered on BADBIR, and 95% of UK dermatology centers prescribing biologics for psoriasis contribute data to BADBIR. To maximize inclusivity, generalizability, and sample size, we developed a prespecified research protocol with inclusion criteria designed to capture a truly representative patient sample. This approach, together with the real-world nature of the cohort necessitating pragmatic sampling, introduces heterogeneity into our dataset that reflects our strategy to maximize inclusivity and minimize selection bias.

In terms of potential limitations, the validity of the therapeutic range is limited to within 1 year of the start of treatment, because this was the selected cohort duration. Most patients in the UK receive the licensed dose for adalimumab (40 mg every 2 weeks), so although dose escalation would be a logical clinical strategy for individuals with subtherapeutic drug levels, this requires confirmation in a clinical trial setting and would have pharmacoeconomic implications. On the other hand, the advent of adalimumab biosimilars at a fraction of the cost of the original drug, means that dose optimization strategies remain highly relevant. Another potential limitation is use of pragmatic serum sampling at routine clinic visits; to account for the timing of samples we included time from last dose as a covariate, and although this was not significant at the univariate level, we only had these data available on around a third of samples. Nevertheless, we identified a range comparable to that of Menting et al. (2015), who reported on trough drug levels, suggesting that limiting sampling to trough levels may not be an absolute requirement. Finally, covariates are not always consistent across outcomes or datasets because of statistical artefacts when using different subsets of patients—thus, our findings require replication. Indeed, the model fit as measured by pseudo *R*^2^ indicates that although drug level is important, this model could potentially be improved using covariates that have not been considered in this study.

## Conclusions

We provide evidence to support the proactive measurement of drug levels in the management of psoriasis with adalimumab therapy. Drug levels taken both early and at steady state during the treatment course could be used to predict and therefore optimize clinical outcome. These findings are of potential relevance to other IMIDs.

## Materials and Methods

### Ethics approval

The study was conducted in the spirit of the 1996 International Conference on Harmonisation in Good Clinical Practice (ICH-GCP) 1996 and in accordance with the 2008 Declaration of Helsinki. The study protocol was approved by The South East London REC 2 Ethics Committee (11/H0802/7). Written informed consent was obtained from all subjects before enrolment.

### Patients and setting

BSTOP is a prospective, multicenter (n = 60) observational study for establishing clinically relevant markers of outcomes to systemic therapies in people with severe psoriasis. All adults in the UK fulfilling the BSTOP inclusion criteria (BSTOP protocol available at https://bit.do/BSTOPDOCS) and enrolled onto BADBIR (http://www.badbir.org/) were invited to participate. BADBIR has recruited more than 12,000 psoriasis patients since 2007 and is unique worldwide in terms of size and depth of phenotyping. Inclusion criteria include dermatologist’s diagnosis of psoriasis; age older than 16 years; and started taking, or switched to, a conventional systemic therapy or a biological therapy within the previous 6 months. Detailed information is recorded, including demographics, comorbidities, treatments, and adverse effects. Clinical response is assessed longitudinally using the criterion standard assessment tool, the PASI.

### Pharmacokinetic measurements

Venous blood samples were collected between June 2009 and December 2016 during routine clinic reviews; samples from some BSTOP patients were taken between 2009 and 2011 as part of a pilot study with the same inclusion criteria. Samples were taken without reference to treatment administration (i.e., trough/nontrough not specified) and immediately centrifuged at 2,000*g* for 10 minutes, and serum aliquots were frozen at –80 °C. In this pragmatic study, samples were not collected from every patient at every time point. Samples within the first year of treatment (n = 961, maximum = 4 samples/patient) were selected and sent in batches to Sanquin (Amsterdam, The Netherlands) for measurement of adalimumab concentration (ELISA, μg/ml [Menting et al., 2015]) and ADA (radioimmunoassay, ADA positive cutoff > 12 arbitrary units/ml [Menting et al., 2015]).

### Outcome measures

Primary treatment response was defined as achieving PASI75; secondary outcomes were (i) PASI90 and (ii) an absolute measure of response, PASI of 1.5 or less (see results in Supplementary Materials online). Baseline PASI was defined as the most recent PASI recorded before the date of the first drug dose within the preceding 6 months (Iskandar et al., 2015, Warren et al., 2015).

### Statistical methods

Analyses for PASI75 and PASI90 responses were restricted to patients with baseline PASI greater than 10 as an accepted criterion for severe disease (National Institute for Health and Care Excellence, 2018) and to minimize confounding due to pre-biologic treatments—of particular relevance in this real-world dataset.

#### Identification of therapeutic range

The therapeutic range dataset (Figure 1, Figure 2) included samples that were taken at steady state (≥9 weeks [Awni et al., 2003]), with PASI scores recorded on the same day. Empirical receiver operating characteristic curve analysis (Menting et al., 2015) was used to identify the lower limit of the therapeutic range—specifically, the drug level at which responders are detected with a minimum sensitivity of 80%.

A descriptive concentration effect curve (Menting et al., 2015) was generated to confirm that clinical response plateaus beyond a certain drug level. Multivariable mixed effects logistic regression was then used to identify an upper drug level and to explore the relationship between drug level and treatment response in the presence of other relevant covariates. A random intercept term was used to account for correlation between repeated samples on the same patient. Univariate mixed effects logistic regression models explored the relationship between treatment response and (i) drug level(ii) other confounding covariates including ADA, time from last dose, sex, age, and disease duration. For continuous covariates, the best-fitting simple nonlinear transformation was chosen based on reduction in the Akaike Information Criterion. Covariates associated with response at significance level *P* < 0.1 were taken forward to the multivariable modeling stage. Forward selection techniques were then used, with covariate inclusion based on significance level *P* < 0.05. Pseudo *R*^2^ (McFadden for fixed effect models, conditional and marginal for mixed models [Nakagawa and Schielzeth, 2013]) and Akaike Information Criterion were calculated to assess model fit. Finally, an upper limit of the therapeutic range was defined based on this multivariable model for PASI75, with the target probability of response set at 80%.

#### Using drug level to predict subsequent response

To investigate whether drug level predicts subsequent outcome, two further datasets were derived: an early dataset comprising samples taken between 1 and 12 weeks with a corresponding PASI 6 months (122–243 days) after start of treatment and a steady state dataset comprising samples taken at steady state (≥9 weeks [Awni et al., 2003]) with a corresponding PASI 6 months (122–243 days) after the sample date. Multivariable mixed effects logistic regression models were considered to explore the relationship between drug level and other covariates with patient response 6 months later. For the early dataset, a random effect was not included because of the small number of patients with multiple samples. All analyses were carried out using Stata, version 14 (StataCorp, 2015) on a complete case basis.

## ORCIDs

Teresa Tsakok: http://orcid.org/0000-0003-1895-6070

Catherine Smith: https://orcid.org/0000-0001-9918-1144

## Conflict of Interest

CEMG has received honoraria and/or research grant support (University of Manchester) from AbbVie, Almirall, Bristol Meyers Squibb, Celgene, GSK, Janssen, LEO Foundation, Lilly, Novartis, Pfizer, Sandoz, Sun Pharma, and UCB Pharma. NJR has received honoraria, travel support, and/or research grants (Newcastle University) from AbbVie, Almirall, Amgen, AstraZeneca, Bristol-Myers Squibb, Celgene, Genentech, Janssen, Leo-Pharma Research Foundation, Novartis, Pfizer, and Stiefel GSK. JB has received honoraria, travel support, and/or research grants (King’s College) from AbbVie, Pfizer, Novartis, Janssen, Roche, Regeneron, Lilly, UCB, Sun Pharma, Boehringer Ingelheim, and GSK. RBW has received honoraria and/or research grants from AbbVie, Almirall, Amgen, Boehringer Ingelheim, Celgene, Janssen, Leo, Lilly, Novartis, Pfizer, Sanofi, Xenoport, and UCB. ADB has received honoraria from AbbVie, Amgen, Boehringer Ingelheim, Celgene, Janssen, Leo, Lilly, Novartis, and Pfizer. TR has received honoraria for lectures from Pfizer, AbbVie, and Regeneron and a research grant from Genmab. DS has received departmental research funding from AstraZeneca. CS has received departmental research funding from AbbVie, GSK, Pfizer, Novartis, Regeneron, and Roche. NW acts as statistician on a trial funded by AstraZeneca. The PSORT consortium has a number of industry partners; see www.psort.org.uk. No other relationships or activities that could appear to have influenced the submitted work.
