# Supplementary material for: Defining the Therapeutic Range for Adalimumab and Predicting Response in Psoriasis: A Multicenter Prospective Observational Cohort Study
Source: J Invest Dermatol. 2019 Jan;139(1):115–23. doi: 10.1016/j.jid.2018.07.028 (PMC6300405; doi:10.1016/j.jid.2018.07.028)

Supplementary document for “Developing a therapeutic range and predicting response to biologics in patients with severe psoriasis: a multi-centre prospective observational cohort study”

Supplementary Table S1: Summary statistics for patients not providing serum samples during the first 12 months on adalimumab

Supplementary Table S2: Diagnostic accuracy and ROC curve cutpoints (therapeutic range dataset)

Supplementary Table S3: Univariate analyses of response (therapeutic range dataset)

Supplementary Table S4: Final multivariable models for PASI90 and PASI≤1.5 response based on drug level and additional covariates (therapeutic range dataset, early dataset and steady state dataset)

Supplementary Table S5: Univariate analyses of 6-month response (early dataset)

Supplementary Table S6: 4-week (21-35 days) drug levels predicting 6-month response (PASI75)

Supplementary Table S7: Univariate analyses of 6-month response (steady state dataset)

Supplementary Figure S1: Probability of (a) PASI90 response (b) PASI≤1.5 response based on drug level taken on the same day (therapeutic range dataset)

Supplementary Figure S2: Probability of 6-month (a) PASI75 response (b) PASI90 response (c) PASI≤1.5 response based on early drug level (early dataset)

Supplementary Figure S3: Probability of 6-month (a) PASI75 response (b) PASI90 response (c) PASI≤1.5 response based on drug level at steady state (steady state dataset)

Supplementary Table S1: Summary statistics for patients not providing serum samples during the first 12 months on adalimumab

	Full cohort (n=409 patients)	
**Covariate**	**Mean (sd)**	**Complete data n (%)**
Baseline PASI	13.9 (8.4)	345 (84.4)
Height (cm)	170.4 (11.0)	356 (87.0)
Weight (kg)	89.2 (21.5)	373 (91.2)
Waist (cm)	100.5 (16.1)	373 (91.2)
BMI (kg/m^2)	30.8 (7.3)	350 (85.6)
Age (years)	44.4 (11.7)	409 (100.0)
Disease Duration (years)	23.4 (11.5)	366 (89.5)
	**n (%)**	
Ethnicity – white	370 (90.5)	409 (100.0)
Gender – male	245 (59.9)	409 (100.0)
Inflammatory arthritis	93 (24.6)	378 (92.4)
Ever smoked	212 (56.1)	378 (92.4)
Palm psoriasis	60 (15.9)	378 (92.4)
Biologic naive	268 (65.5)	409 (100.0)

Supplementary Table S2: Diagnostic accuracy and ROC curve cutpoints (therapeutic range dataset)

	PASI90		PASI≤1.5	
**Cutpoint**	3.2	7	3.2	7
**Sensitivity**	82.46%	40.35%	84.84%	45.13%
**Specificity**	41.18%	75.21%	36.90%	70.99%
**Overall classification accuracy**	58.44%	60.64%	57.91%	59.65%
**Positive Predictive Value**	50.18%	53.91%	51.20%	54.82%
**Negative Predictive Value**	76.56%	63.70%	75.72%	62.38%
**AUC (95% CI)**	0.65 (0.60,0.70)		0.64 (0.60,0.68)	
**Response rate: all samples**	41.81%		43.83%	
**Response rate: samples with drug level < cutpoint ***	23.44%	36.30%	24.28%	37.62%
**Response rate: samples with drug level ≥ cutpoint ***	50.18%	53.91%	51.20%	54.82%

Note: analyses are based on 409 samples from 303 patients for PASI90 and 632 samples on 454 patients for PASI≤1.5, due to the rule that the baseline PASI should be greater than 10.
* Response rates for samples above and below cutpoints are equivalent to positive predictive value and (1 – negative predictive value) respectively.

Supplementary Table S3: Univariate analyses of response (therapeutic range dataset)

	PASI75			PASI90			PASI≤1.5		
**Covariate**	**Coef (s.e)**	**OR (95% CI)**	**P value**	**Coef (s.e)**	**OR (95% CI)**	**P value**	**Coef (s.e)**	**OR (95% CI)**	**P value**
Sqrt(drug level)	1.08 (0.20)	2.94 (1.97,4.37)	<.001	0.85 (0.20)	2.34 (1.59,3.45)	<.001	0.90 (0.17)	2.46 (1.75,3.45)	<.001
Detect ADA	-1.29 (0.32)	0.28 (0.15,0.51)	<.001	-0.36 (0.33)	0.69 (0.36,1.32)	.27	-0.75 (0.29)	0.47 (0.27,0.83)	.010
Disease duration (years)	-0.01 (0.01)	0.99 (0.97,1.02)	.51	-0.02 (0.01)	0.98 (0.95,1.01)	.13	-0.01 (0.01)	0.99 (0.97,1.02)	.55
BMI	-0.06 (0.02)	0.94 (0.90,0.98)	.004	-0.08 (0.03)	0.92 (0.87,0.97)	.003	-0.06 (0.02)	0.94 (0.90,0.98)	.007
Gender - male	0.41 (0.29)	1.51 (0.86,2.65)	.15	-0.23 (0.35)	0.79 (0.40,1.59)	.52	-0.76 (0.31)	0.47 (0.25,0.86)	.015
Baseline PASI	0.03 (0.02)	1.03 (0.98,1.08)	.23	0.08 (0.03)	1.08 (1.02,1.16)	.01	-0.01 (0.02)	0.99 (0.94,1.03)	.59
Height	-0.01 (0.01)	0.99 (0.97,1.02)	.72	-0.02 (0.02)	0.98 (0.95,1.01)	.26	-0.03 (0.02)	0.97 (0.94,1.00)	.044
Weight	-0.02 (0.01)	0.98 (0.96,0.99)	.002	-0.03 (0.01)	0.97 (0.95,0.99)	.001	-0.03 (0.01)	0.97 (0.96,0.99)	.001
Waist	-0.03 (0.01)	0.97 (0.95,0.99)	.007	-0.05 (0.01)	0.95 (0.93,0.98)	.001	-0.05 (0.01)	0.95 (0.93,0.98)	<.001
Inflammatory arthritis	-0.12 (0.33)	0.89 (0.47,1.69)	.72	-0.29 (0.40)	0.75 (0.34,1.65)	.48	0.06 (0.33)	1.07 (0.56,2.02)	.85
Ever smoked	-0.15 (0.28)	0.86 (0.49,1.50)	.59	-0.68 (0.36)	0.51 (0.25,1.02)	.057	-0.50 (0.30)	0.60 (0.34,1.09)	.092
Age	-0.004 (0.011)	1.00 (0.97,1.02)	.74	-0.01 (0.01)	0.99 (0.96,1.02)	.43	-0.01 (0.01)	0.99 (0.97,1.02)	.58
Treatment duration (days)	0.001 (0.001)	1.000 (0.998,1.004)	.57	0.004 (0.002)	1.004 (1.000,1.007)	.029	0.002 (0.001)	1.002 (0.999,1.004)	.19

Time of sample from last dose (days)	-0.003 (0.069)	1.00 (0.87,1.14)	.96	-0.07 (0.06)*	0.93 (0.83,1.05)	.20	0.01 (0.05)	1.01 (0.92,1.11)	.78
Palm psoriasis	-0.48 (0.37)	0.62 (0.30,1.26)	.19	-0.71 (0.47)	0.49 (0.20,1.24)	.13	-0.82 (0.40)	0.44 (0.20,0.96)	.039
Ethnicity - white	1.07 (0.45)	2.90 (1.20,6.99)	.018	0.62 (0.58)	1.86 (0.60,5.75)	.29	0.77 (0.49)	2.16 (0.83,5.62)	.11
Biologic naive	0.37 (0.33)	1.45 (0.48)	.25	0.99 (0.44)	2.68 (1.13,6.34)	.025	1.11 (0.32)	3.02 (1.62,5.66)	.001

A random effect has been included in the models to allow for multiple samples per patient. Covariates with $p < 0.1$ are taken forward to multivariable modelling step with the exception of “BMI” and “Waist” due to correlation with the preferred covariate “Weight”.

Supplementary Table S4: Final multivariable models for PASI90 and PASI≤1.5 response based on drug level and additional covariates (therapeutic range dataset, early dataset and steady state dataset)

**Therapeutic range dataset** (Mixed effects logistic regression models)								
	**Covariate**	**Coefficient (s.e)**	**P value**	**95% CI**	**OR (95% CI)**	**Marginal / Conditional pseudo R^2**	**Number of samples**	**Number of responders (% of samples)**
PASI90	Sqrt(drug level)	1.08 (0.23)	<.001	(0.62,1.53)	2.93 (1.86,4.64)	0.21/0.58	409 samples on 303 patients	171 (41.81)
	Treatment duration (days)	0.006 (0.002)	.002	(0.002,0.009)	1.01 (1.00,1.01)			
	Baseline PASI	0.10 (0.03)	.004	(0.03,0.17)	1.10 (1.03,1.18)			
	Biologic naive	1.00 (0.45)	.025	(0.13,1.87)	2.71 (1.13,6.50)			
PASI≤1.5	Sqrt(drug level)	0.90 (0.17)	<.001	(0.57,1.23)	2.45 (1.77,3.41)	0.16/ 0.55	632 samples on 454 patients	277 (43.83)
	Gender - male	-0.77 (0.30)	.009	(-1.36,-0.19)	0.46 (0.26,0.83)			
	Biologic naive	1.11 (0.31)	<.001	(0.50,1.73)	3.05 (1.65,5.63)			
**Early dataset** (Logistic regression models)								
	**Covariate**	**Coefficient (s.e)**	**P value**	**95% CI**	**OR (95% CI)**	**Pseudo R^2**	**Number of samples**	**Number of responders (% of samples)**
PASI90	Sqrt(drug level)	1.16 (0.28)	<.001	(0.62,1.71)	3.20 (1.86,5.51)	0.12	159 samples on 120 patients	71 (44.65)
	Ethnicity - white	1.20 (0.55)	.031	(0.11,2.28)	3.30 (1.12,9.77)			
PASI≤1.5	Sqrt(drug level)	0.72 (0.19)	<0.001	(0.34,1.10)	2.05 (1.41,2.99)	0.09	225 samples on 177 patients	114 (50.67)
	BL PASI	-0.06 (0.02)	0.006	(-0.11,-0.02)	0.94 (0.90,0.98)			

**Steady state dataset** (Mixed effects logistic regression models)								
	**Covariate**	**Coefficient (s.e)**	**P value**	**95% CI**	**OR (95% CI)**	**Marginal / Conditional pseudo R^2**	**Number of samples**	**Number of responders (% of samples)**
PASI90	Sqrt(drug level)	1.11 (0.32)	.001	(0.48,1.74)	3.04 (1.62,5.72)	0.14/0.73	322 samples on 244 patients	130 (40.37)
	Palm psoriasis	-1.65 (0.76)	.030	(-3.14,-0.16)	0.19 (0.04,0.85)			
PASI≤1.5	Sqrt(drug level)	1.48 (0.33)	<.001	(0.83,2.13)	4.41 (2.30,8.45)	0.16/0.81	500 samples on 370 patients	212 (42.40)
	Biologic naive	1.38 (0.57)	.016	(0.26,2.51)	3.98 (1.29,12.26)			

Supplementary Table S5: Univariate analyses of 6-month response (early dataset)

	PASI75			PASI90			PASI≤1.5		
**Covariate**	**Coef (s.e)**	**OR (95% CI)**	**P value**	**Coef (s.e)**	**OR (95% CI)**	**P value**	**Coef (s.e)**	**OR (95% CI)**	**P value**
Sqrt(drug level)	0.95 (0.26)	2.59 (1.56,4.32)	<.001	1.08 (0.27)	2.96 (1.76,4.98)	<.001	0.72 (0.18)	2.05 (1.43,2.93)	<.001
Detect ADA	-1.05 (0.45)	0.35 (0.14,0.85)	.020	-1.02 (0.50)	0.36 (0.13,0.96)	.041	-1.37 (0.43)	0.25 (0.11,0.59)	.001
Disease duration (years)	-0.04 (0.02)	0.96 (0.93,0.99)	.016	-0.04 (0.02)	0.96 (0.93,0.99)	.010	-0.01 (0.01)	0.99 (0.97,1.02)	.56
BMI	-0.07 (0.03)	0.94 (0.89,0.99)	.011	-0.10 (0.03)	0.91 (0.86,0.96)	<.001	-0.08 (0.02)	0.93 (0.89,0.97)	.001
Gender - male	0.71 (0.36)	2.02 (0.99,4.13)	.053	0.52 (0.35)	1.68 (0.84,3.35)	.14	0.19 (0.27)	1.21 (0.71,2.06)	.49
Baseline PASI	0.02 (0.03)	1.02 (0.96,1.08)	.56	-0.03 (0.03)	0.97 (0.92,1.03)	.34	-0.07 (0.02)	0.93 (0.89,0.97)	.002
Height	0.01 (0.02)	1.01 (0.97,1.04)	.76	0.01 (0.02)	1.01 (0.98,1.05)	.46	0.01 (0.01)	1.01 (0.98,1.04)	.55
Weight	-0.02 (0.01)	0.98 (0.96,1.00)	.031	-0.03 (0.01)	0.97 (0.96,0.99)	.002	-0.02 (0.01)	0.98 (0.97,0.99)	.002
Waist	-0.02 (0.01)	0.98 (0.96,1.01)	.17	-0.02 (0.01)	0.98 (0.95,1.00)	.023	-0.03 (0.01)	0.97 (0.96,0.99)	.005
Inflammatory arthritis	0.32 (0.44)	1.38 (0.58,3.27)	.46	-0.47 (0.38)	1.60 (0.75,3.39)	.22	0.45 (0.33)	1.57 (0.83,2.98)	.16
Ever smoked	-0.52 (0.38)	0.60 (0.28,1.26)	.18	-0.02 (0.34)	0.98 (0.51,1.88)	.94	-0.29 (0.26)	0.75 (0.45,1.26)	.27
Age	-0.004 (0.014)	1.00 (0.97,1.02)	.77	-0.02 (0.01)	0.98 (0.95,1.00)	.11	-0.01 (0.01)	0.99 (0.97,1.01)	.58

Treatment duration (days)	-0.004 (0.007)	1.00 (0.98,1.01)	.55	-0.002 (0.006)	1.00 (0.99,1.01)	.80	-0.003 (0.005)	0.997 (0.987,1.008)	.60
Time of sample from last dose (days)	0.01 (0.09)	1.01 (0.86,1.20)	.88	0.06 (0.08)	1.07 (0.92,1.24)	.41	0.02 (0.06)	1.02 (0.91,1.14)	.73
Palm psoriasis	0.005 (0.466)	1.00 (0.40,2.50)	>.99	-0.46 (0.43)	0.63 (0.27,1.47)	.29	-0.22 (0.32)	0.81 (0.43,1.52)	.51
Ethnicity - white	0.81 (0.47)	2.25 (0.90,5.65)	.084	0.88 (0.51)	2.41 (0.89,6.52)	.084	0.41 (0.39)	1.50 (0.71,3.20)	.29
Biologic naïve	0.22 (0.42)	1.25 (0.55,2.84)	.59	-0.04 (0.39)	0.96 (0.44,2.07)	.92	0.41 (0.29)	1.51 (0.86,2.66)	.15

Covariates with $p < 0.1$ are taken forward to multivariable modelling step with the exception of “BMI” and “Waist” due to its correlation with the preferred covariate “Weight”.

Supplementary Table S6: Very early (4-week) drug levels predicting PASI75 response at 6 months

	Covariate	Coefficient (s.e)	P value	95% CI	OR (95% CI)	Pseudo R^2	Number of samples	Number of responders (% of samples)
**PASI75**	**Sqrt(drug level)**	1.76 (0.74)	0.017	(0.31,3.21)	5.83 (1.37,24.85)	0.18	49 samples on 47 patients	37 (75.51)
	**Ethnicity – white**	2.07 (1.12)	0.065	(-0.13,4.28)	7.96 (0.88,72.14)			

A multivariable logistic regression model has been fitted with the covariates drug level and ethnicity.

Supplementary Table S7: Univariate analyses of 6-month response (steady state dataset)

	PASI75			PASI90			PASI≤1.5		
**Covariate**	**Coef (s.e)**	**OR (95% CI)**	**P value**	**Coef (s.e)**	**OR (95% CI)**	**P valu e**	**Coef (s.e)**	**OR (95% CI)**	**P value**
Sqrt(drug level)	1.02 (0.21)	2.78 (1.83,4.24)	<0.001	1.14 (0.33)	3.12 (1.64,5.96)	.001	1.51 (0.33)	4.54 (2.36,8.72)	<.001
Detect ADA	-1.57 (0.47)	0.21 (0.08,0.52)	0.001	-1.33 (0.58)	0.27 (0.08,0.84)	.023	-2.14 (-3.38,0.89)	0.12 (0.03,0.41)	.001
Disease duration (years)	-0.005 (0.017)	1.00 (0.96,1.03)	0.79	-0.03 (0.02)	0.97 (0.93,1.02)	.23	-0.02 (0.02)	0.98 (0.94,1.02)	.38
BMI	-0.09 (0.03)	0.92 (0.86,0.98)	0.012	-0.12 (0.05)	0.89 (0.81,0.98)	.020	-0.13 (0.05)	0.88 (0.80,0.96)	.005
Gender - male	0.49 (0.46)	1.64 (0.66,4.06)	0.29	-0.11 (0.58)	0.90 (0.29,2.82)	.85	-0.96 (0.54)	0.38 (0.13,1.12)	.079
Baseline PASI	0.02 (0.04)	1.02 (0.94,1.09)	0.68	0.09 (0.05)	1.09 (0.98,1.21)	.12	-0.04 (0.04)	0.96 (0.89,1.04)	.30
Height	0.01 (0.02)	1.01 (0.97,1.05)	0.68	-0.003 (0.027)	1.00 (0.95,1.05)	.91	-0.02 (0.02)	0.98 (0.93,1.03)	.39
Weight	-0.03 (0.01)	0.97 (0.95,0.99)	0.014	-0.04 (0.02)	0.96 (0.02)	.026	-0.04 (0.02)	0.96 (0.93,0.99)	.005
Waist	-0.04 (0.02)	0.96 (0.93,0.99)	0.006	-0.06 (0.02)	0.94 (0.90,0.99)	.011	-0.07 (0.02)	0.93 (0.90,0.97)	.001
Inflammatory arthritis	0.24 (0.48)	1.28 (0.50,3.29)	0.61	0.34 (0.66)	1.40 (0.39,5.11)	.61	0.89 (0.58)	2.44 (0.78,7.61)	.13
Ever smoked	-0.23 (0.44)	0.80 (0.34,1.87)	0.60	-0.81 (0.60)	0.45 (0.14,1.45)	.18	-0.62 (0.51)	0.54 (0.20,1.46)	.23
Age	-0.003 (0.017)	1.00 (0.96,1.03)	0.87	-0.01 (0.02)	0.99 (0.94,1.03)	.51	-0.03 (0.02)	0.97 (0.93,1.01)	.12

Treatment duration (days)	-0.0004 (0.0019)	1.000 (0.996,1.003)	0.83	-0.003 (0.003)	0.997 (0.992,1.002)	.21	-0.003 (0.002)	1.00 (0.99,1.00)	.098
Time of sample from last dose (days)	-0.11 (0.12)	0.90 (0.71,1.15)	0.39	-0.09 (0.12)	0.91 (0.72,1.15)	.44	-0.02 (0.05)*	0.98 (0.90,1.07)*	.64
Palm psoriasis	-0.71 (0.57)	0.49 (0.16,1.50)	0.21	-1.74 (0.78)	0.18 (0.04,0.81)	.025	-1.19 (0.68)	0.30 (0.08,1.14)	.078
Ethnicity - white	1.03 (0.66)	2.80 (0.77,10.20)	0.12	1.00 (0.86)	2.73 (0.50,14.85)	.25	0.04 (0.78)	1.04 (0.23,4.76)	.06
Biologic naive	0.69 (0.52)	2.00 (0.73,5.51)	.18	1.25 (0.71)	3.48 (0.86,14.00)	.079	1.42 (0.57)	4.15 (1.36,12.63)	.012

A random effect has been included in the models to allow for multiple samples per patient. Covariates with p < 0.1 are taken forward to multivariable modelling step with the exception of “BMI” and “Waist” due to correlation with the preferred covariate “Weight”. * Standard logistic regression with no random effect was used due to convergence issues.

Supplementary Figure S1: Probability of (a) PASI90 response (b) PASI≤1.5 response based on drug level taken on the same day (therapeutic range dataset)

(a) Probability of PASI90 response based on drug level for biologic naive patients (yellow line) and non-biologic naive patients (bright blue line). The green dots are the proportion of PASI90 responders for the groups of patients in the concentration effect curve in Figure 2b of the main paper. The probabilities are marginal predicted means due to the inclusion of treatment duration, baseline PASI and a random effect in the model.

(b) Probability of PASI≤1.5 response based on drug level for biologic naive patients (yellow line) and not biologic naive (bright blue line). The green dots indicate the proportion of PASI≤1.5 responders for the groups of patients in the concentration effect curve in Figure 2b of the main paper. The probabilities are marginal predicted means due to the inclusion of gender and a random effect in the model.

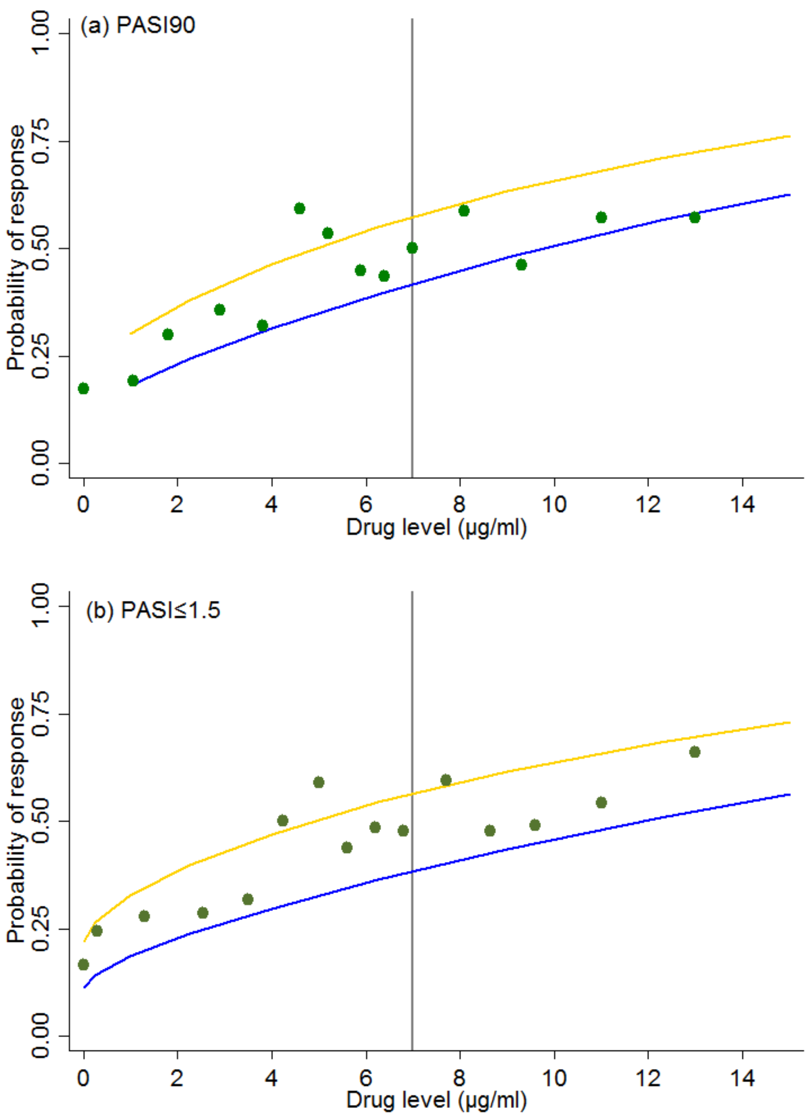

Supplementary Figure S2: Probability of 6-month (a) PASI75 response (b) PASI90 response (c) PASI≤1.5 response based on early drug level (early dataset)

(a) Probability of PASI75 response based on early drug level for white ethnicity (red line) and all other ethnicities (dark blue line).

(b) Probability of PASI90 response based on early drug level for white ethnicity (red line) and all other ethnicities (dark blue line).

(c) Probability of PASI≤1.5 response based on early drug level.

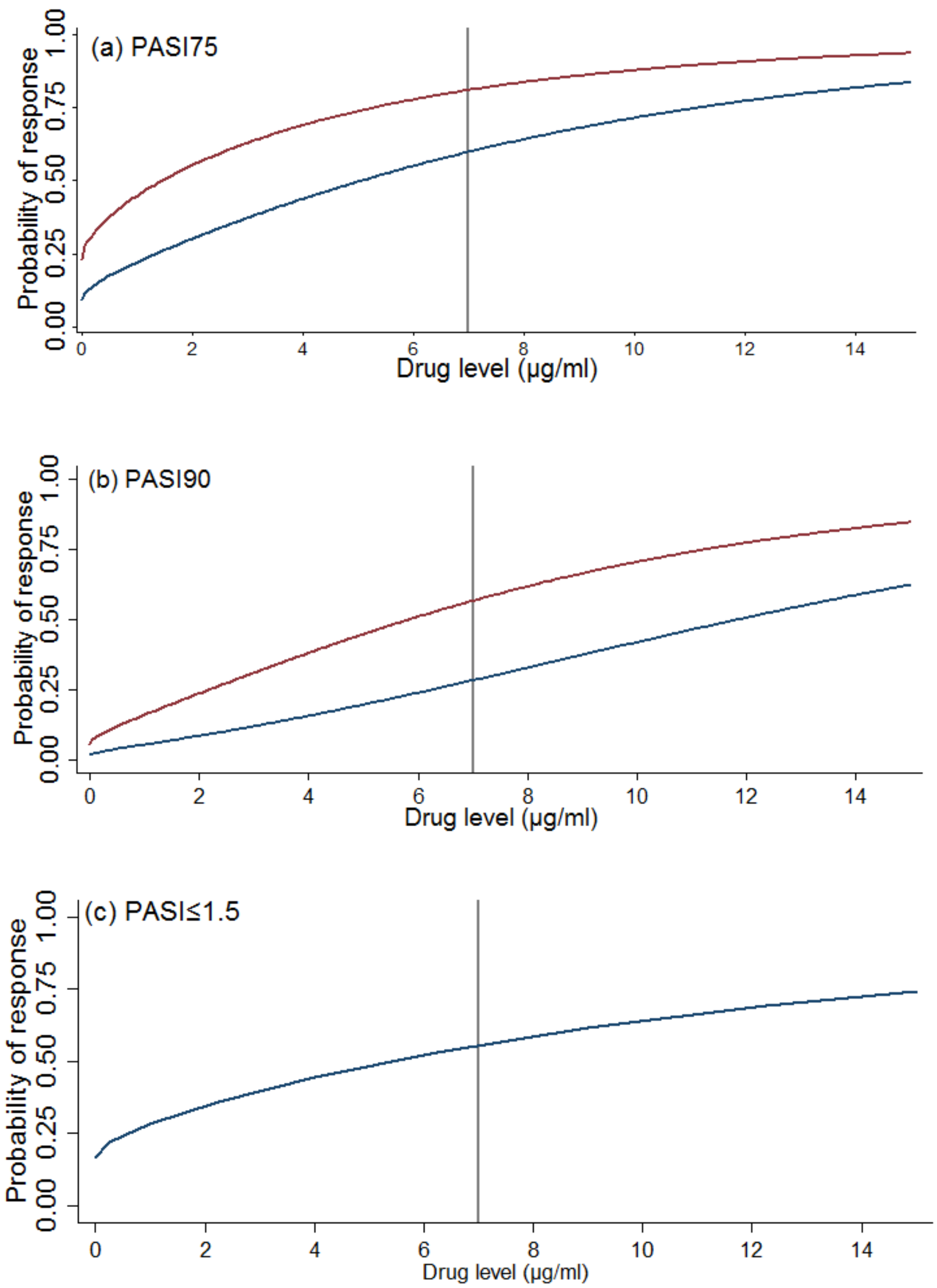

Supplementary Figure S3: Probability of 6-month (a) PASI75 response (b) PASI90 response (c) PASI≤1.5 response based on drug level at steady state (steady state dataset)

(a) Probability of PASI75 response based on drug level at steady state.

(b) Probability of PASI90 response based on drug level at steady state.

(c) Probability of PASI≤1.5 response based on drug level at steady state for biologic naive patients (yellow line) and non-biologic naive patients (bright blue line). The probabilities are marginal predicted means due to the inclusion of a random effect in the model.

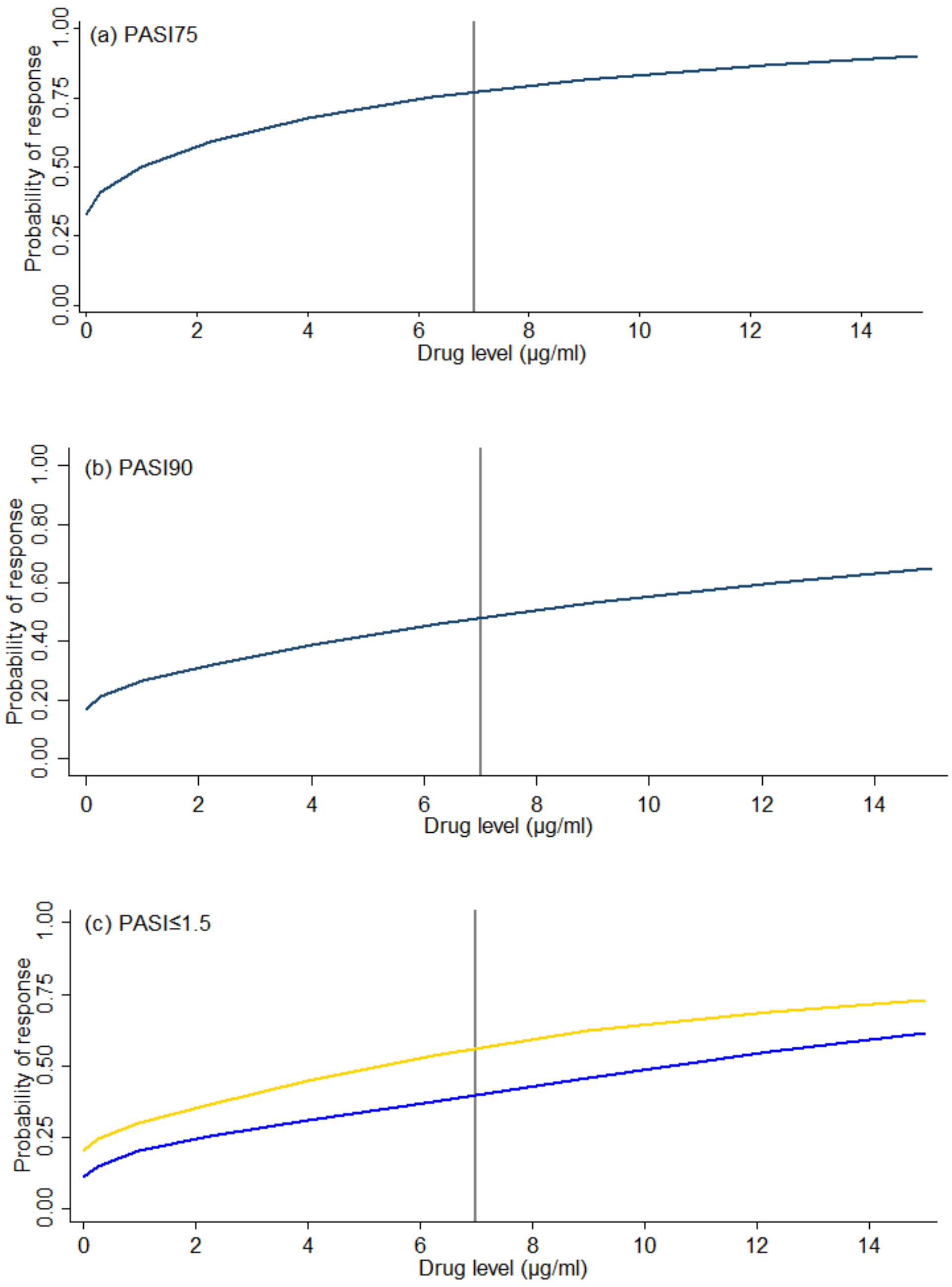

Supplement: Supplementary Data [file mmc1.pdf]
